# The SARS-CoV-2 pandemic: remaining uncertainties in our understanding of the epidemiology and transmission dynamics of the virus, and challenges to be overcome

**DOI:** 10.1098/rsfs.2021.0008

**Published:** 2021-10-12

**Authors:** Roy M. Anderson, Carolin Vegvari, T. Déirdre Hollingsworth, Li Pi, Rosie Maddren, Chi Wai Ng, Rebecca F. Baggaley

**Affiliations:** ^1^ Department of Infectious Disease Epidemiology, Imperial College London, London, UK; ^2^ Big Data Institute, Li Ka Shing Centre for Health Information and Discovery, University of Oxford, Oxford, UK; ^3^ Joint Universities Pandemic and Epidemiological Research (JUNIPER) consortium, University of Leicester, Leicester, UK; ^4^ Department of Respiratory Sciences, University of Leicester, Leicester, UK

**Keywords:** COVID-19, SARS-CoV-2, epidemiology, mass vaccination, herd immunity, transmission

## Abstract

Great progress has been made over the past 18 months in scientific understanding of the biology, epidemiology and pathogenesis of SARS-CoV-2. Extraordinary advances have been made in vaccine development and the execution of clinical trials of possible therapies. However, uncertainties remain, and this review assesses these in the context of virus transmission, epidemiology, control by social distancing measures and mass vaccination and the effect on all of these on emerging variants. We briefly review the current state of the global pandemic, focussing on what is, and what is not, well understood about the parameters that control viral transmission and make up the constituent parts of the basic reproductive number *R*_0_. Major areas of uncertainty include factors predisposing to asymptomatic infection, the population fraction that is asymptomatic, the infectiousness of asymptomatic compared to symptomatic individuals, the contribution of viral transmission of such individuals and what variables influence this. The duration of immunity post infection and post vaccination is also currently unknown, as is the phenotypic consequences of continual viral evolution and the emergence of many viral variants not just in one location, but globally, given the high connectivity between populations in the modern world. The pattern of spread of new variants is also examined. We review what can be learnt from contact tracing, household studies and whole-genome sequencing, regarding where people acquire infection, and how households are seeded with infection since they constitute a major location for viral transmission. We conclude by discussing the challenges to attaining herd immunity, given the uncertainty in the duration of vaccine-mediated immunity, the threat of continued evolution of the virus as demonstrated by the emergence and rapid spread of the Delta variant, and the logistics of vaccine manufacturing and delivery to achieve universal coverage worldwide. Significantly more support from higher income countries (HIC) is required in low- and middle-income countries over the coming year to ensure the creation of community-wide protection by mass vaccination is a global target, not one just for HIC. Unvaccinated populations create opportunities for viral evolution since the net rate of evolution is directly proportional to the number of cases occurring per unit of time. The unit for assessing success in achieving herd immunity is not any individual country, but the world.

## 1. Introduction

A catalogue of the number of deaths induced by the major epidemics of historical times dwarfs the total deaths on all past battlefields. However, until the emergence of the coronavirus SARS-CoV-2 epidemic at the end of 2019, the world's population today had not witnessed the rapid emergence of a directly transmitted respiratory tract infection with a high case fatality rate (CFR), which quickly became a global pandemic. Case morbidity and fatality rates from this viral infection remain a great cause for concern and continue to challenge the healthcare resources of many countries in both high-income countries (HIC) and low- and middle-income countries (LMIC), as novel, faster spreading variants emerge. The only comparison in recent times, but still beyond the memory of even the oldest age groups, is the 1918 flu pandemic which caused at least 50 million deaths worldwide. This was the so-called ‘mother of all pandemics' [1]. For comparison, by mid-August 2021, with 206 million cases reported worldwide, estimates of the mortality caused by SARS-CoV-2 are of the order of 4.3 million [2]. Undoubtably, the numbers reported are underestimates both of cases, due to asymptomatic infection and suboptimal testing, and of mortalities, due to limited reporting systems of the cause of death in many countries.

The past four decades have witnessed a series of major infectious disease outbreaks that have caused much suffering and loss of life. Some have turned into pandemics with no country spared, such as that of the HIV-1 virus, the aetiological agent of AIDS. UNAIDS estimates that by the end of 2020 approximately 30–40 million people have died of AIDS since the start of the pandemic in the 1980s. This is roughly an average of 1 million per year [3] which is less than that caused by SARS-CoV-2 during the 2020 year. Others such as epidemic of SARS-CoV-1 virus transmission (generally referred to as the SARS outbreak) in 2003, were limited by the inability of the virus to replicate in its new human host, such that symptoms of infection occurred before most patients were infectious to others. As such, simple quarantining and isolation effectively controlled spread [4]. Similar methods worked for the Ebola epidemics of 2013–2016 in West Africa and the Democratic Republic of Congo in 2018–2020. Both of these outbreaks, although very significant locally and of unprecedented magnitude in historical terms for Ebola due to its spread to urban areas and increased mobilization across borders, never became pandemics, as infection was restricted to few countries' specific localities [5]. Although having a very high infection fatality rate (IFR), Ebola could be controlled by good contact tracing and isolation procedures. The 2009 H1N1 influenza pandemic lasted for about 19 months, although the magnitude of the pandemic was initially overestimated, as, in fact, the CFR was lower than a typical seasonal influenza A strain. This was rectified once the correct denominator was employed to include the many infections that were typically very mild, especially in children [6]. The Zika virus, transmitted by mosquitoes, was a further recent epidemic of significance. In October 2015, Brazil reported an association between Zika virus infection and microcephaly. Outbreaks and evidence of transmission soon appeared throughout the Americas, Africa and other regions of the world. To date, a total of 86 countries and territories have reported evidence of mosquito-transmitted Zika infection. Although a cause of serious morbidity, no effective vaccine has been developed and reported infections to the World Health Organization (WHO) fell to very low levels by January 2020. In many respects, the epidemic has not attracted the attention it deserves, perhaps because its impact in HIC has been limited [7]. This may change as global warming influences the distribution of important insect vectors of viral and protozoan infections.

Eighteen months from its recorded start in January 2020 (the real start date is thought to be as early as October 2019 [8]), the SARS-CoV-2 pandemic is far from over, with extensive recent spread of the Delta variant in Asia. The future remains uncertain, yet there is a high likelihood the virus will become endemic globally, with patterns of seasonal incidence, given the likely short duration in infection- and vaccine-mediated immunity and possible animal reservoirs. This is despite great progress in vaccine development and manufacture, understanding of the epidemiology of this virus, tracking its evolution, determining its possible zoonotic origins and clinical research on how best to manage severe infection in patients. Most notably, within 12 months from the overt beginning of the epidemic, three vaccines were approved for use in the general population by regulators in Europe and North America at the beginning of 2021 [9].

Four features of the coronavirus make its rapid spread very difficult to control by conventional public health measures such as detection and patient isolation. These are: (i) a high direct rate of transmission from person to person via inhaled microdroplets when the infected person coughs, sneezes, speaks, sings or breathes (contaminated surfaces are now thought to be less important [10]), (ii) a high fraction of asymptomatic infections, (iii) a period of infectiousness of perhaps 1–2 days in length before clear symptoms of infection develop in those who eventually become symptomatic and (iv) long incubation and infectious periods (the former having an average value of around 5 days and the both may last for 14 days in some individuals [11]). All these features are believed to vary in quantitative detail by SARS-CoV-2 variant, although information is limited for most of the more recently identified variants such as the Delta variant.

Despite our increased knowledge, there remain important gaps in our understanding of the biology, evolution, clinical epidemiology and transmission dynamics of the virus. This review examines what is, and what is not, well understood about the epidemiology, transmission dynamics and control of SARS-CoV-2. We focus on five areas: namely, the current global pattern of the epidemic, key epidemiological parameters that determine the transmission dynamics of the virus and how these may vary by SARS-CoV-2 variant, model structures of viral transmission that are employed to predict future trends in the epidemic, information on ‘who infects whom’ and the challenges surrounding the creation of herd immunity by mass vaccination.

## 2. Current state of the pandemic

The spread of the virus up to mid-2021 continues worldwide despite high vaccine uptake in some countries, and strongly enforced measures in many countries to diagnose infection, trace contacts, impose travel restrictions and non-pharmaceutical interventions (NPIs) such as social distancing and mask-wearing [10]. These NPIs are currently the only truly effective way to limit spread in the absence of both high levels of herd immunity created by mass vaccination in most countries, and effective treatments of cases to reduce infectiousness to others. In many countries, a major surge in cases was reported in late 2020 and early 2021, due to a combination of effects including the emergence of more transmissible variants such as Delta, variable compliance to social distancing measures, widely varying vaccination levels country by country and the seasonal effects of winter in the Northern Hemisphere influencing human behaviour and activity.

The most striking feature of the pandemic over the past 18 months has been the continued and rapid evolution of the virus, with multiple distinct variants in circulation, all with slightly or markedly different epidemiological and pathogenesis properties. The major SARS-CoV-2 variants in circulation at the time of writing, mid-2021, are B.1.1.7 (Alpha variant—originally identified in Kent in the UK), B.1.351 (Beta variant—originally identified in South Africa), P.1 (Gamma variant—originally identified in Brazil), B.1.429 (Epsilon variant—first detected in California, USA), B.1.526 (Iota variant—first reported in New York, USA) and B.1.617.2 (Delta variant—first identified in India) in order of appearance from the start of the pandemic. Many others have been identified and designated as variants of concern (VOC), variants of interest or variants under monitor by WHO and governmental public health agencies. Some countries and regions list up to 22 genetic variants of interest or concern as illustrated by the European Centres for Disease Control (ECDC). The spread of the highly contagious Delta variant first identified in India in December 2020 has been extensive in recent months and is of particular concern. It swept rapidly through India and the UK before reaching the US, SE Asia and, most recently, China. It is now the dominant variant in many countries due to its very high basic reproductive number (*R*_0_) which is thought to be at least double that of the highest measured value of all the other identified ancestral variants [12]. Concerns arising from this emerging picture of viral evolution are primarily the implications on protection conferred by current vaccines, and secondly the longer term implications for vaccine development and modification. The experiences in China in early 2020 revealed most effective way to limit the spread of a novel and highly transmissible, directly transmitted, viral infection in the absence of a vaccine or antiviral therapies, was attempting to stop contact between people outside of a household via social distancing measures, or ‘lockdown’. By imposing very strict rules on the movement and mixing of the population, the rapidly growing epidemic in January 2020 was quickly turned over, and cases fell to very low levels for the rest of the year. More recently, China has reported frequent and widespread outbreaks of the highly transmissible Delta variant and in response is now reimposing strict bans on mixing and travel.

**Figure 1.**
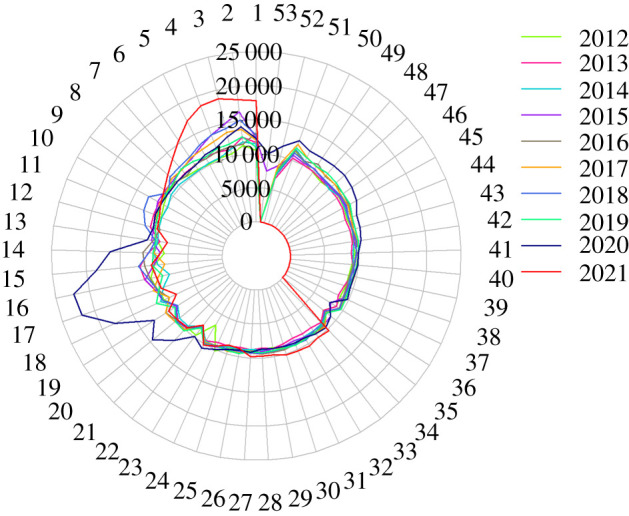
Radial plot (‘Florence Nightingale’ plot) of excess deaths in England and Wales by year and week number (1–53). Data from the Office for National Statistics (ONS) during the pre-pandemic years 2012–2019 and during the pandemic in England & Wales [15]. Data for 2021 were only recorded up to week 34 at the time of plotting the Figure. Therefore, the deaths in weeks 35–52 in 2021 are zero.

Recently, a sero-epidemiological survey from Wuhan, China, where the virus first came to international attention, suggests that reported COVID-19 cases numbers are far lower, perhaps by a factor 10 than the number with antibodies to the new coronavirus [13]. Wuhan reported 50 345 cases by the end of December 2020, yet 4.43% seroprevalence in a population of 11 million people suggests 487 300 people have been infected. This may be due to a high fraction of asymptomatic cases and/or poor reporting of cases in the early stages of the epidemic. Similar to Wuhan, in most countries, case reporting does not provide an adequate picture of the true extent of viral spread. The scale of national testing programmes has a great influence on reported case numbers. The data from the National Health Service (NHS) Test and Trace programme in England provides a good example. When introduced in May 2020, the weekly numbers tested were less than 500 000, while in mid-December 2020 the number had risen to above 1.5 million [14]. Different countries and organizations put differing weights on various measures of the spread and impact of the virus. These include case numbers, serological surveys, contact tracing, hospitalizations, deaths thought to be due to COVID-19 and excess mortality when judged against pre-pandemic years. All have strengths and weaknesses depending on resource availability and the quality of the public health infrastructure. Arguably, the most accurate assessment of the state of the epidemic in terms of viral spread and the impact of control measures such as mass vaccination in a given country, is provided by data on excess mortality week by week when compared with recent pre-pandemic data (figure 1).

**Figure 2.**
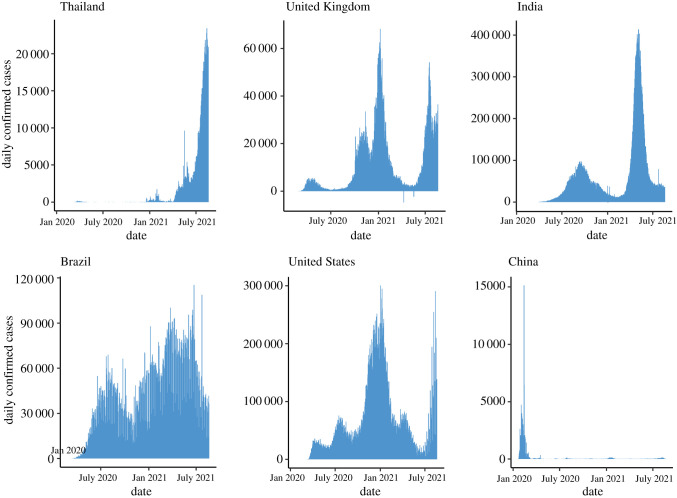
Patterns in reported SARS-CoV-2 cases per week in Thailand, United Kingdom, India, Brazil, US and China, up to 20 August 2021 illustrating the great heterogeneity in the pattern of the epidemic in different countries (source: Johns Hopkins University, USA [2]).

The experiences of China stimulated most countries to impose NPIs of varying degrees of severity, and at varying time points in the growth of the first wave of the epidemic in early 2020. Prevailing opinion at the start of the pandemic, that countries could not minimize both deaths and the economic impact, led to prioritizing mortality reductions, albeit with some delays in lockdown implementation in some instances. Governments needed to implement measures to ameliorate the inevitable economic downturn [16,17]. It was also made clear that there were difficult decisions ahead for governments. How individuals respond to advice on how best to prevent transmission would be as important as government actions, if not more important. Government communication strategies to keep the public informed of how best to avoid infection would be vital, as would extra support to manage the economic downturn. Early analyses of the possible course of the epidemic and the consequences of relaxing mitigation measures have been shown to be largely accurate. Delays in implementing mitigation measures and the advantages of the early introduction of lockdown actions are well illustrated by comparing the course of the epidemic in Thailand and the UK (figure 2). Despite being the first country to report a COVID-19 case outside China, Thailand was successful in containing spread in 2020 through timely implementation of NPIs, strictly enforced [18], in contrast to the delay in implementing lockdown in the UK. The current surge of cases in Thailand reflects the spread of Alpha and Delta variants in the region; public health measures successful during the first wave in 2020 have so far proved unsuccessful [19]. The virtual absence of strong government action or messaging on the importance of social distancing mitigation measures in 2020 is also well illustrated by the patterns of the epidemic observed, for example, in the USA and Brazil (figure 2). The situation in the USA at the beginning of 2021 was alarming, with deaths due to infection exceeding deaths of USA military in the Second World War, and with patients requiring hospitalization as a result of infection exceeding capacity in many cities. At present, in mid-2021, infection has again risen to high levels, despite good vaccine uptake in many states, due to the establishment of the Delta variant. Interestingly, with the benefit of hindsight, the perceived trade-off between public health and economic impact has been questioned: the delayed imposition of national lockdowns in countries such as the USA and UK elevated numbers of infections and deaths, ultimately requiring longer, more stringent NPIs causing more severe economic consequences [20].

A clear example of the impact of social distancing measures, plus the effect of relaxing such measures, is provided by case data from the UK (figure 2). Note the slow decay of the epidemic after an initial peak once *R_t_* < 1, and its resurgence once measures were relaxed over the summer months of 2020 resulting in *R_t_* > 1 in many regions of the UK. The limited impact of the second lockdown in late autumn is also apparent, which along with seasonal effects on viral transmission and the emergence of a more transmissible viral variant (Beta), necessitated the third lockdown in early January 2021. There now exist social distancing indices such as Oxford University's COVID-19 Government Response Tracker [21], which reports measures including a containment and health index, combining lockdown restrictions with other measures such as testing policies and contact tracing, and applications such as the Numerus Model Builder [22], designed to measure the intensity and impacts of interventions including social distancing. Such tools are intended to allow decision-makers and citizens to understand government responses in a consistent way and to assess the impacts of various interventions for themselves.

Mass vaccination is beginning to have a major impact on the pattern of the epidemic both in infections recorded, but most importantly on hospitalizations and deaths, as seen in countries such as Malta, Iceland, USA, Singapore and Israel. The most striking feature of the different global patterns is primarily the great heterogeneity, part of which will be the reliability of data on reported cases, especially in LMIC where resources for public health are limited. Second, where countries did well initially in controlling transmission, spread is now extensive in large part due to the arrival of the highly transmissible Delta variant. Third, the beneficial impact of vaccination in those countries which have received adequate supplies, and who can afford vaccine purchase on scale. Finally, where NPIs have been implemented, and advice from governments followed by the respective populations, transmission of the virus has been controlled well until measures have been relaxed as a direct consequence of the high economic impact.

Regions such as Taiwan and New Zealand, who developed and implemented good contact tracing systems at very early stages of viral spread, with high compliance to quarantine and isolation measures (applied to case contacts and international arrivals), stand as the best examples of effectively controlling viral spread [23,24]. The predictions of a second wave of infection in the early autumn in the Northern Hemisphere, post the relaxation of social distancing measures in the summer, proved to be true [25]. The magnitude of the basic reproductive number, *R*_0_, was sufficiently high (between 2 and 4 for the ancestral variants [26]) to ensure continued and effective viral transmission (*R*_0_ > 1) through both winter and summer months. This is very different from seasonal influenza A, where *R*_0_ values typically fall below unity in value in the summer months in Northern Hemisphere countries [27].

In many European countries, social distancing measures introduced to curtail the growth of the second wave in the autumn of 2020 have not been as successful as those put in place to limit the size of the first wave in March and April. A number of factors may be involved, including the season (winter being a time of closer contacts in enclosed spaces, whether at the workplace, on transport or in the household), different stringencies of restrictions, communities tiring of restrictions on movement and mixing and viral evolution leading to the establishment of more transmissible variants of SARS-CoV-2 such as the Alpha and Delta variants.

## 3. Viral evolution

Ongoing whole viral genome sequencing studies show continual viral evolution with mutations accumulating in key genes such as that encoding for the spike protein that is used to gain entry into host cells when compared with early viral genome sequences (the ancestral variants) collected at the beginning of 2020 [28]. This is to be expected for RNA coronaviruses, but it is important to note that many mutations will not necessarily influence the phenotype.

**Figure 3.**
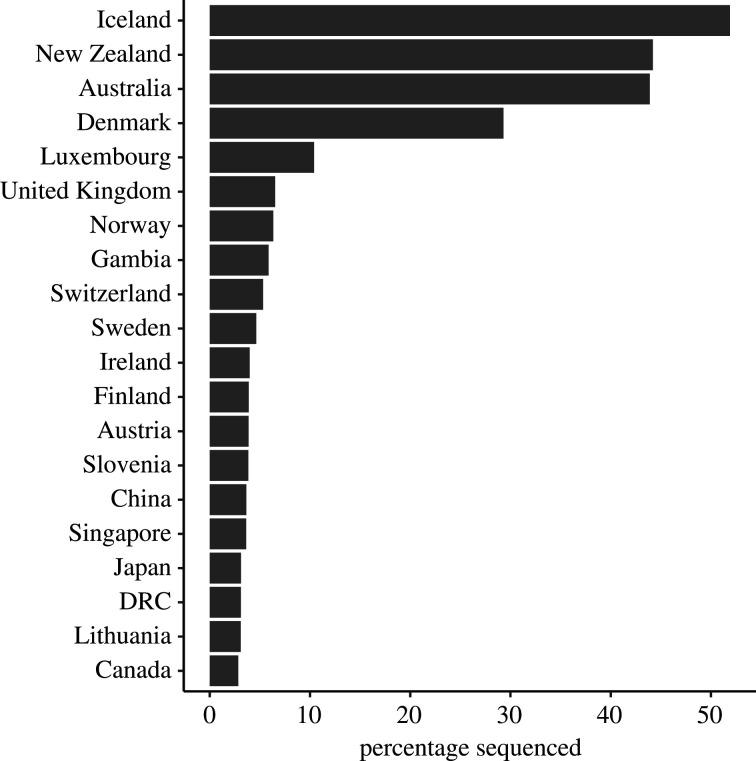
The percentage of cases reported in which sequence information of the virus is acquired by country. Only the 20 countries with the highest frequencies are shown (data source: Johns Hopkins University and Medicine Coronavirus Resource Center [30].

The UK has provided much important information on viral evolution through the activities of the COVID-19 Genomics UK (COG-UK) consortium, which was created to deliver large-scale and rapid whole-genome virus sequencing to local health centres and the UK government (electronic supplementary material, figure S1). An example of the surveillance picture of variant frequency in the UK in August 2021 is given in table 1 for data from June 2021. Note that of those viral samples sequenced, the Alpha variant is still very prevalent, but the Delta variant showed the most rapid rise in frequency over the time period highlight in the table. Other countries have followed the UK's example, and high volumes of sequence data are being collected worldwide. The information in table 1 illustrates the complexity of the pandemic in any given country, with many variants cocirculating, and the obvious difficulties in predicting future trends when each of these variants may have different phenotypic properties. Of importance in assessing such data and hence the pattern of viral evolution and spread, is the fraction of sequences performed in relation to the number of reported cases in a country. Data on the fraction sequenced of reported cases are presented in figure 3 and show that most countries only sequence a very small fraction of all cases recorded. Much more sequencing is required to effectively track viral evolution globally.

**Table 1.** Summary of variants of concern (VOC) and variants under investigation (VUI) in August 2021 in the United Kingdom (source: Public Health England [29]).

variant^a^	alternative names	lineage	total confirmed sequencing and genotyping cases, to 1 August 2021	new cases since 4 August 2021
Alpha	VOC-202012/01	B.1.1.7	272 927	245
Beta	VOC-202012/02	501Y.V2 B.1.351	1090	3
Delta	VOC-21APR-02	B.1.617.2 AY.1 and AY.2	433 079	51 057
VOC-21FEB-02	VOC02-202102/	B.1.1.7 with E484 K	46	0
Gamma	VOC-202101/02	P.1	263	3
Kappa	VUI-21APR-01	B.1.617.1	495	1
VUI-21APR-03	n.a.	B.1.617.3	14	0
VUI-21FEB-01	VUI-202101/02	A.23.1 with E484 K	79	0
Eta	VUI-202102/03	B.1.525 (previously designated UK1188)	495	0
VUI-21FEB-04	VUI-202102/03	B.1.1.318	350	4
Zeta	VUI-202101/01	P.2	60	0
Theta	VUI-21Mar-02	P.3	10	3
VUI-21MAY-01	n.a.	AV.1	185	0
VUI-21MAY-02	n.a.	C.36.3	153	0
Lambda	VUI-21JUN-01	C.37	8	0
VUI-21JUL-01	n.a.	B.1.621	40	3

^a^Using WHO naming where available.

Genotypic changes are relatively easy to chart. The associated phenotypic changes, if any, are more difficult to establish since they involve longitudinal studies of changes in the frequency of the variant and associated changes in host immune responses, plus epidemiological characteristics of infection with the virus. Our knowledge of the differences in key properties of each variant is very limited at the time of writing, mid-2021. For example, our knowledge of variant-specific incubation and infectious periods and their distributions, clinical outcomes and if any cross-protection results from immunological responses to conserved regions of the viral genome, is very poor.

Relatively small differences in the magnitude of *R*_0_ by variant can create a situation where the small fitness advantage (reflected by the magnitude of *R*_0_) in a novel variant, results in the rapid replacement of the lower fitness variant. A deterministic simulation of the effect of two variants spreading in the same population, where one has a slightly bigger *R*_0_ (electronic supplementary material, figure S2), illustrates how even a very small advantage can lead rapidly to variant dominance over a period of just a few months (as observed by the B.1.1.7 lineage spread in England in late 2020, electronic supplementary material, figure S1). Changes in frequency of variants show a sigmoid-shaped curve rising to either complete dominance (frequency of 1, electronic supplementary material, figure S2) or coexistence (see table 3 in Anderson & May [31]).

**Figure 4.**
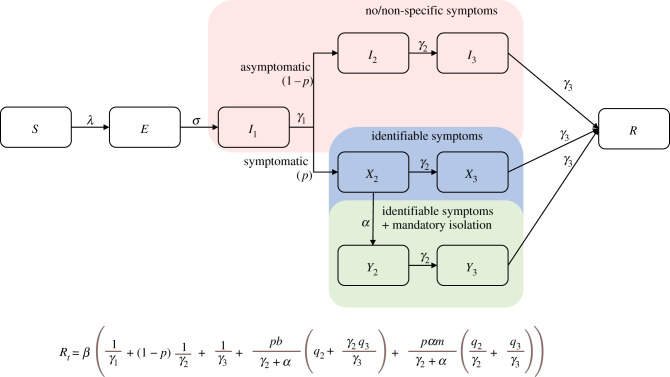
The flow chart of a simple epidemic model for COVID-19 of individual states and pathways representing rates of transfer between states [25]. The top pathway is for the fraction (1 − *p*) of those infected who are asymptomatic, with no symptoms or non-specific, unrecognized symptoms, who stay in the community and eventually recover—but contribute to transmission. The bottom pathway is for the fraction (*p*) who have identifiable symptoms who either self-isolate (minimal level of behaviour change, reducing the infection rate by (1 − *b*)) or get admitted to hospital (mandatory self-isolation/hospitalization, which is assumed to be far more effective, reducing the infection rate by (1 − *m*)). I_1_ is the pre-symptomatic stage, during which individuals are infectious before the *p* fraction develop symptoms. Two phases are added to produce an Erlang distribution for time to symptoms. No social distancing or lockdown is represented in the diagram. Below the flow chart is the *R_t_* equation that arises from this flow chart. It is defined as an effective reproduction number because self-imposed or mandatory isolation is assumed to take place. In this equation 1/*α* is the average number of days it takes from symptom onset to isolation. *λ* is the force of infection term: the rate at which susceptible individuals become infected, which is dependent on the infection rates (expected amount of people an infected person infects per day) for each infectious state in the model. The *β* term is the infection rate for the pre-symptomatic infectious state, while *q*_2_ and *q*_3_ are the relative increases in infection rate for symptomatic individuals in phases 2 and 3 of infection, respectively. The *γ* terms define rates of leaving a given state (1/*γ* is the average duration of stay). See electronic supplementary material, Information for the expression of *λ* and derivation of *R_t_*.

A fitness advantage is defined by increased transmission (*R*_0_) and this can be achieved in a variety of ways since several epidemiological parameters determine the magnitude of *R*_0_ [32]. It may or may not be related to increased virulence, as reflected in the CFR [33]. Higher viraemia leading to greater infectiousness is one possibility, as is a shorter incubation period implying rapid growth in viraemia, such that the generation time of the new variant is shorter than other variants in circulation, plus some combination of a series of effects on the key components of *R*_0_ (figure 4).

There is growing evidence that the Delta variant has a similar or shorter incubation period but higher viral loads than the ancestral variants [34]. The study conducted by Li *et al.*, in China of recent spread of the Delta variant, based on daily sequential polymerase chain reaction (PCR) testing of quarantined subjects, indicated the viral load of the first positive test of Delta infections was approximately 1000 times higher than that of the 19A/19B variant infections back in the initial epidemic wave of 2020, suggesting a potentially faster viral replication rate and higher infectiousness of the Delta variant at an early stage of infection. Few such studies exist of other novel variants and much more work of this type is required to better understand the relative *R*_0_ values of different variants and how the various components of *R*_0_ vary. Transmission is not the only property of interest, clearly, pathogenicity in terms of requiring hospitalization and concomitant mortality, plus impact on the efficacies of the different vaccines in wide use, are very important and again very poorly understood at present.

A survey in the UK published in August 2021 provided some important information on the effectiveness of two most widely used vaccines (Pfizer-BioNTech and Oxford-AstraZeneca). The research looked at the effectiveness of the vaccines comparing the Delta variant (the most common variant in the UK since mid-May) and the Alpha variant (the most common variant in the UK in the previous six months). It found that vaccinations are still effective in preventing new infections compared with unvaccinated individuals, but the vaccines are less effective against the Delta variant compared to the Alpha variant. While the risk of getting COVID-19 is still lower for people who had received two doses of either vaccine if they do get COVID-19 they seem to have just as much virus in their nose and throat as people who have not been vaccinated. They, therefore, may be able to transmit the infection [35].

## 4. Key epidemiological parameters and remaining uncertainties

The major processes determining the transmission dynamics of SARS-CoV-2 are depicted diagrammatically in a flow chart in figure 4. These all influence the magnitude of *R_t_* as described by an expression for the effective reproductive number (effective not basic since isolation is represented in the flow chart), immediately below the flow chart [16,25]. Each of these processes, such as the incubation and infectious periods, is distributed and virtually all are influenced by confounding variables that include age, gender, ethnicity, social and cultural factors, comorbidities, and time plus spatial location. The complex and composite nature of the many factors that influence the magnitude of *R*_0_ and *R_t_* well illustrate how many parameters could be influenced by mutations in the viral genome that could in turn impact transmissibility.

What should be measured to track the course of the epidemic within a country or defined location, and how reliable are such measures given existing data sources? Some countries, such as the UK, report both the magnitude of the effective reproduction number, *R_t_*, which describes the average number of secondary cases generated by primary cases at time *t*, and the epidemic growth rate, *r_t_*, which describes the rate of change in case numbers over a defined time period [11,36]. Both are stratified by area in the country. Other countries rely on case reports, deaths, hospital admissions or excess mortality to chart the epidemic over time, with the expectation that any social distancing measures put in place will decrease these numbers albeit with a significant time delay, especially for deaths.

The value of *r_t_* is easier to estimate using simple statistical methods on changes in incidence over time. If negative in value, the epidemic is contracting, and *R_t_* < 1 which is the goal for stopping transmission. *R_t_* is a more informative epidemiological measure, although measurement requires assumptions of other epidemiological parameters, such as the generation time (average time from infection to passing on to secondary cases) that can change over the course of the epidemic and is known to vary by variant. Much attention has focused on the magnitude of *R_t_* at time *t*, labelled by the media as the *R* number or rate. It is not a rate, but a dimensionless parameter made up of many constituent parts [32] that include clinical epidemiological details of the typical course of infection in an individual, behavioural and demographic factors that influence transmission, and the impact of any imposed control measures. Many sources of variability exist and there is much uncertainty around some of the key epidemiological processes that determine the magnitude of *R_t_* [25] (figure 4). These include the fraction of infections that are asymptomatic, how infectious asymptomatic infections typically are, and the duration of the infectious period before symptoms appear. Also of importance is the probability distribution of the generation of secondary cases, which is over-dispersed (variance > mean) such that most infected individuals transmit none or a few infections, and a few individuals transmit many—the so-called ‘super-spreaders’ [37]. Further complicating matters is variation in many of the parameter components that make up *R*_0_ by variant. This issue is currently very poorly understood.

Sources of data for the estimation of *R_t_* and *r_t_* and how they change over time, include reported case numbers, serological surveys, data from contact tracing and COVID-19 deaths. The specificity and sensitivity of the PCR and lateral flow tests for detecting active viral infection, and the serological tests for detecting the presence of antibodies, are key for interpreting data. PCR tests detect dead virus as well as live virus. This may be behind cases that test positive weeks after initial infection. However, at the moment it is not clear if these positive results are due to the continued presence of dead virus or if SARS-CoV-2 can establish prolonged infection, especially in immunocompromised individuals. Continued assessment of the accuracy of all tests is essential because of genetic heterogeneity in the SARS-CoV-2 genome at sites that might form the target of the PCR amplification process, and the period over which neutralizing and other antibodies to viral antigens can be detected. Evidence has emerged showing decaying antibody titres to viral antigen over time periods of 100 days post seroconversion [38]. A large range of SARS-CoV-2-neutralizing antibody titres has been reported after infection and these vary depending on the length of time from infection and the severity of disease [39]. However, the neutralizing antibody titre required for protection from reinfection and/or disease in humans is not yet understood.

### 4.1. The basic reproductive number, *R*_0_

A review of published estimates of *R*_0_ based on data from the early stages of the epidemic in various countries with ancestral variants of the virus by the Royal Society SET-C committee [25] reported values between 2 and 4+. This review also documents the distribution of *R_t_* values based on contact tracing data and household studies, showing overdispersion with the variance greater than the mean value (using a negative binomial probability of this distribution, the aggregation parameter *k* is in the range 0.1–0.8, although data remain limited at the time of writing, mid-2021). A recent systematic review from Imperial College London on secondary attack rates identified 45 studies providing data on spread and the generation of secondary cases in household settings [40]. Household studies do provide valuable information, but they may only constitute part of the total number of cases generated by an index case since some transmission events will be outside the defined household.

As mentioned earlier, the value of the intrinsic transmission potential of the virus, as measured by *R*_0_, is now known to vary by variant. Evidence regarding a new variant, Alpha, identified in the UK late in 2020 (VUI-202012/01) suggested a competitive advantage over the existing variants in circulation, implying a higher *R*_0_ value, perhaps as much as 40% higher [41]. Estimates are available for *R_t_*, both for the Alpha variant and those variants circulating previously in the same populations, but comparative calculations need to take into account seasonality, since all variants will be transmitted more efficiently in the winter months due to people aggregating more in enclosed settings with less air circulation than in the summer months. Most recently, data are emerging on the properties of the Delta variant that has spread so rapidly throughout large regions of the world during the mid-part of 2021. The basic reproductive number is estimated to be of the order of 5–6 or more in some populations, with a shorter incubation period and much higher infectiousness, as indicated by very high viral load in infected patients [12].

### 4.2. Incubation period

Published data on the incubation period of SARS-CoV-2, the average time from infection to the appearance of symptoms of infection (in those who develop symptoms), has, recently, been reviewed [11]. The published data suggest average values of around 5–6 days, where the distribution of individual values has a long right-hand tail (the variance is greater than the mean in value). The gamma and lognormal distributions have both been shown to provide a good fit to data from reasonably large samples of patients. The importance of this parameter is somewhat less than usual for SARS-CoV-2, given that a high fraction of infections may be asymptomatic. This topic is addressed under a separate heading. Seroconversion in hospitalized patients is typically 50% by day 7, and in all patients by day 14 [42]. The incubation period of the Delta variant appears to be shorter, but data are currently very limited [41,42].

### 4.3. Generation time and serial intervals

The generation time, *T*, for an infectious disease is the time between infection events in an infector–infectee pair of individuals. Serial intervals describe the average time between symptoms of infection in the transmitter to when the person he or she infects, develops symptoms. In conjunction with estimates of *R*, the generation time can provide insights into the speed of COVID-19 spread, driven by the profile of infectiousness over time (see below). It is difficult to measure directly as it is hard to ascertain time of infection since it is usually unobserved. There are far more estimates of the serial interval because it is much easier to record [11]; these are then often used as a proxy of the former. However, ignoring the difference between the serial interval and generation time can lead to biased estimates of *R*.

After the chance events at the beginning of the epidemic (when case numbers are small and reporting unreliable) are over, the cases of infection (or a measure of this statistic) grow exponentially until herd immunity or control measures move *R* to less than unity in value. At this early stage, the instantaneous *r* of the exponentially growing epidemic curve is approximately given by

$$r=\frac{R_{0}-1}{T}.$$

This equation gives a link between the value of *R*_0_ and the speed with which infection spreads from one person to the next in chains of transmission. Both *R*_0_ and *T* determine *r*, but *R*_0_ dominates the area under the unmitigated epidemic curve and hence the total number of cases, and *T* greatly influences the timescale of the epidemic's growth and decay. Note that as a statistic, the control of transmission by mitigation measures requires the value of *r* to be less than zero [11]. As mentioned above, it is easier to measure the serial interval as symptom onset is easier to identify than time of infection acquisition. This value is often used interchangeably with the generation time since it is easier to measure via contact tracing studies. However, in the case of SARS-CoV-2 it has less relevance given that many infections, especially in the young, do not seem to generate marked and easily identifiable symptoms. Some studies suggest that between 5% [43–45] and 80% [46] of infected people do not show clear symptoms of infection. This very wide range depends on many confounding variables such as age, gender, location, the existence of other predisposing medical conditions and study design. As reviewed in a Royal Society SET-C report, typical estimates of the COVID-19 serial interval range between 3 and 6 days in the early stages of epidemics in most countries with good contact tracing systems [11]. Little is known of this parameter when stratified by variant, although recent research suggests no significant change compared with other variants [47].

Typical values of the generation time at the start of a COVID-19 epidemic (where the value of the effective reproductive number *R_t_* is close to *R*_0_) are between 3 and 7 days [11]. Values of both the generation time and the serial interval vary over the course of an epidemic, lengthening as the magnitude of *R_t_* declines from its pristine *R*_0_ value. Data on how this relationship changes over the course of an epidemic in a location are currently limited. This is of importance since methods for estimating *R_t_* often depend on a prior assumption about the magnitude of either the generation time or the serial interval [11,48,49], or a prior assumption about the value of *R*_0_ when estimating the generation time.

### 4.4. Infectious period and infectiousness

Much remains uncertain about infectiousness and the duration of this state and how it varies among variants. Its study is complicated by continued viral evolution. SARS-CoV-2 infects cells in both the upper respiratory tract (URT) and the lower respiratory tract (LRT) [50,51]. It enters host cells via the receptor ACE-2 (angiotensin-converting enzyme 2) on epithelial cell surfaces. Structural analysis suggests that variants isolated early in the pandemic of SARS-CoV-2 bind to the receptor greater than 10-fold more efficiently than SARS-CoV-1, partially explaining the comparatively high contagiousness of the virus. Early analyses of the new alpha variant of SARS-CoV-2, VUI-202012/01 (based on sequencing 126 219 viral genomes) suggest that the N501 mutation in the region of the spike protein (the receptor-binding domain that the virus uses to bind to the human ACE-2 receptor on epithelial cells) improves binding. This is believed to result in much more efficient entry into cells, leading to faster viral growth and higher viraemia within the human host. This in turn results in higher transmission capabilities (larger variant-specific *R*_0_ values). Studies of viraemia in patients infected with the Delta variant suggest shorter incubation periods and much higher viraemia by comparison with ancestral variants [12].

**Figure 5.**
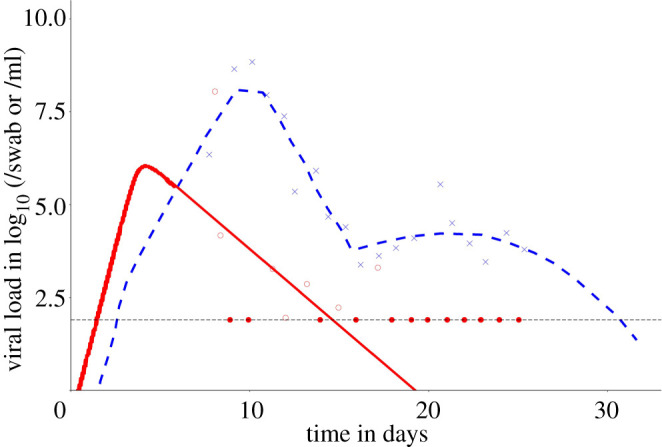
Data from Ke *et al.* [52] describing viraemia in hospitalized patients over time since infection, employing data from Germany in the early stages of the pandemic [42]. The red dots are viral load in the URT and the blue crosses are viral load in the LRT. The dotted horizontal line is the limit of detection. The red and blue lines are fits of the model of viral dynamics within patients. The model assumes target cell limitation and spatial spread of virions within the host. The model was fitted to data points by minimizing the residual sum of squares. For details see Ke *et al.* [52]. The filled dots represent values at the limit of detection.

It is likely that the ability of the virus to effectively infect cells in the URT allows the virus to be transmitted before symptom onset (which occurs an average of 5 days post infection in those who develop symptoms [50,51]). Very few patients may still have detectable viraemia in the URT 10–14 days post infection, and a still smaller fraction may have viraemia in the URT detectable 20 days after infection [52]. This coronavirus thus has a long period of potential infectiousness. An example from a recent study [52] is shown in figure 5, which shows viral load is high at symptom onset on average 5 days after infection, such that significant viral loads in the URT will be present at least 1–2 days prior to symptoms are detected [42].

With respect to infectivity, Wölfel and colleagues attempted live virus isolation at multiple time points from clinical samples. The virus was readily isolated during the first week of symptoms from a considerable fraction of samples (16.7% of swabs and 83.3% of sputum samples), but no isolates were obtained from samples taken after day 8 despite moderate to high viral loads being detected by qPCR. Although unproven currently, it is thought to be highly likely that infectiousness is positively related to viral load in the URT [53]. Viral load and the logarithm of viral load have both been used as surrogates of infectiousness [52].

The high viraemia at symptom onset is a very important characteristic of this viral infection that makes it difficult to control, as it implies high infectiousness before symptom onset. However, at the time of writing, mid-2021, data are lacking on viral growth prior to symptom onset, which is difficult to acquire, since contacts of infected people would have to provide URT and LRT samples from the day of contact, to when symptoms appear, in some subset of the sample of people. While challenging, this research needs to be done.

The reasoning behind the hypothesis that people are infectious before symptoms show (in those becoming symptomatic), rests on analyses of the clinical course of viraemia post symptom onset based on qPCR tests for viral load, typically in hospitalized patients (who may have higher viraemia and severity of symptoms than those not requiring hospitalization).

As noted by Wölfel *et al.* [42], viral load also differs considerably between SARS-CoV-1 and SARS-CoV-2. For SARS-CoV-1, it took 7–10 days after the onset of symptoms until peak RNA concentrations (of up to 5 × 10^5^ copies per swab) were reached [54,55]. In the German study [56] of SARS-CoV-2 in early 2020, peak concentrations were reached before day 5, and were more than 1000 times higher, resulting in much higher transmissibility. Successful isolation of live virus from throat swabs is another notable difference between SARS-CoV-2 and SARS-CoV-1, for which such isolation was rarely successful [42].

### 4.5. Fraction with asymptomatic infection

A particular feature of infection with SARS-CoV-2 is the high fraction of individuals who have no or non-specific symptoms of infection. This fraction is dependent on several confounding variables, such as existing comorbidities, and age especially. The asymptomatic proportion appears much higher in children and young adults compared with those over 50 years of age. A recent review documents these patterns [11] (table 2). Some of the more precise studies are those of outbreaks on ships such as the Diamond Princess cruise ship, wherein a sample of 640 people tested, 18% reported no symptoms (on average an older population than many other examples, generating a lower proportion of asymptomatics) [65]. In an Italian village (Vo' Euganeo), between 50% and 75% reported no symptoms, where the age range was much broader. Studies on naval vessels, populated largely by young adults, reported high proportions asymptomatic, as documented in a recent review [66].

**Table 2.** Selection of reports of the proportion of SARS-CoV-2 infections that are asymptomatic, based on meta-analyses and studies with reasonable sample sizes. The proportion of true asymptomatic cases in the general population is around 15–45% (earlier estimates of around 80% are overestimates because of various biases). Confounding factors include changing understanding of the spectrum of symptoms caused by infection; case definition, especially at the beginning of the incubation period (pre-symptomatic cases); inadequate follow-up and differences between different population sub-groups such as age classes. CI, confidence interval; CrI, credible interval.

study	setting	estimate and distribution	sample size
Buitrago-Garcia *et al.* [57]	meta-analysis of 79 studies in 19 countries or territories	20% (95% CI 17–25%, 95% prediction interval 3–67%)	1287/6616 infected cases
Byambasuren *et al.* [58]	meta-analysis of 13 studies in 7 countries or territories	17% (95% CI 14–20%); higher in aged care (20%; 95% CI 14–27%) than in non-aged care (16%; 95% CI 13–20%) (*p* < 0.05)	111/663 infected cases
Yanes-Lane *et al.* [59]	meta-analysis of 10 studies in 3 countries	obstetric patients: 59% (95% CI 49–68%); nursing home residents: 28% (95% CI 13–50%)	74/97 infected obstetric cases; 71/220 infected nursing home residents
Liu *et al.* [60]	meta-analysis of 29 studies in 4 countries	18.9% (95% CI 12.1–26.6%)	248/1726 infected paediatrics cases in four countries
Beale *et al.* [61]	meta-analysis of 22 studies in 10 countries	28% (95% CI 20–35%; 95% prediction interval 0–62%)	unclear
Pollán *et al.* [62]	Spain, serological study	28.5% (95% CI 25.6–31.6%)	680/2390 infected cases screened with immunoassay
Ng *et al.* [63]	Singapore	36% (95% CrI 27–45%)	29/44 infected cases together with Bayesian modelling for 7770 close contacts
Sah *et al.* [64]	meta-analysis of 170 studies worldwide	35.1% (95% CI: 30.7–39.9%)	7220/25 050 infected cases

Petersen & Phillips examined data from 36 601 individuals tested for the virus, of whom 115 tested positive across a broad age range in the UK [67]. They found 76.5% of their random sample who tested positive reported no symptoms, and 86.1% reported none of the symptoms specific to COVID-19. It was unclear if the asymptomatic infected individuals were infectious to others, or if infectious, if they were less infectious and/or infectious for shorter durations than symptomatic individuals. The major implication of this study is that widespread testing programmes need to focus on these ‘silent’ infections via contact tracing to assess their role in viral spread.

Little is understood at present on the factors that predispose to asymptomatic infection, except the inverse correlation with age. Furthermore, there is not an extreme dichotomy between symptomatic and asymptomatic; rather, there is a clinical spectrum from mild to critical illness, with instances of long COVID occurring after seemingly asymptomatic infection [68] and studies reported high incidence of subclinical computed tomography (CT) changes among COVID-19 cases [69,70]. Predisposition could relate to past coronavirus infections, to the infecting dose (received when exposed), how the immune system changes with age or some combination of factors. Understanding this aspect better is an important priority for the coming year.

### 4.6. Case fatality rates

Three recent systematic reviews have examined the factors associated with more severe infection and mortality [71,72]. The most detailed study of the IFR (i.e. the ratio of fatalities to total infections, as opposed to the CFR the ratio of deaths to reported cases) is a systematic review by Levin *et al.* [73] of 27 studies, focused on age as the key confounding variable.

Authors concluded that the age-specific IFR is very low for children and young adults (e.g. 0.002% at age 10 and 0.01% at age 25), but increases progressively to 0.4% at age 55, 1.4% at age 65, 4.6% at age 75 and 15% at age 85 (see plot of IFR versus age with cases including asymptomatic individuals, electronic supplementary material, figure S3). Analyses suggest that about 90% of the variation in population IFR across geographical locations reflects differences in the age composition of the population and the extent to which relatively vulnerable age groups were exposed to the virus. These results indicate that SARS-CoV-2 is hazardous not only for the elderly but also for middle-aged adults, for whom the IFR is far higher than seasonal Influenza A. They suggest that the overall IFR for SARS-CoV-2 should not be viewed as a fixed parameter but as firmly linked to the age-specific pattern of infections.

A further detailed review of 61 published studies documenting IFR values across LMIC compared with HIC, is that of Ioannidis [74]. It reveals substantial variation between many different settings for an overall rate based on samples of mixed ages. However, age again emerges as the most important determinant [74]. Consequently, public health measures to mitigate infections, such as vaccination, should be focused initially on the most at-risk age groups guided by data on CFRs stratified by age, gender, ethnic group and comorbidities [74].

With respect to variation by viral variant, to date no evidence exists to suggest that the Delta variant causes more serious disease. However, viral load is greater so there is a possibility that pathogenesis may be altered in people infected with this variant.

### 4.7. Duration of immunity and reinfection

The known biology and epidemiology of SARS-CoV-2 has some features not always observed with other directly transmitted viral infections including asymptomatic individuals, who may or may not be infectious to others and a pre-symptomatic infectious period. A ‘known unknown’ is the duration of immunity post recovery. Limited data from other coronavirus infections suggest full immunity to reinfection lasts a matter of months rather than years for SARS-CoV-1 and MERS [11], but it is not clear if those reinfected are again infectious to others or exhibit symptoms of infection that result in measurable morbidity [16,75,76]. There may of course be ‘unknown unknowns’ (unmeasured parameters and unknown pathways of infection, transmission and disease) and more complexity may unfold in the second year of the pandemic, perhaps driven by viral evolution. There are few well-documented cases of reinfection to date, but growing evidence suggests reinfection is perhaps more common in unvaccinated individuals than originally envisaged [77].

The largest longitudinal study to date (called SIREN) of reinfection in healthcare workers reported reinfection to be rare, but the reported 44/6614 participants [0.67%] is significant. Furthermore, the report details a small number of people with antibodies were potentially still able to carry and transmit the virus [78].

There is a wide spectrum in the degree of immunity conferred by viral infection, typically depending on the antigenic variation present in a viral population characterized by the presence of more than one distinct variant, based on the immune responses it elicits. For example, infection with the measles virus almost always results in lifelong immunity due to the antigenic homogeneity of the virus. By contrast, respiratory syncytial virus (RSV) can result in multiple infection episodes in the same winter. This is thought to be due to the circulation of many variants of the virus at any one time, each with distinct antigenic properties. Reinfections with seasonal coronaviruses, such as HCoVNL63, HCoV-229E, HCoV-OC43 and HCoV-HKU1, have been observed as early as six months post infection. Frequent reinfections have been shown from 12 months post infection [79] and evidence suggests that the typical duration of protective immunity for these viruses is 1–3 years [76,79–86]. Infection with SARS-CoV-2 leads to detectable immune responses, but the susceptibility of previously infected individuals to reinfection with SARS-CoV-2 remains poorly understood at this stage in the pandemic. SARS-CoV-2 infection elicits neutralizing antibody generation in patients, but how this translates into protective immunity to subsequent infection with SARS-CoV-2 has yet to be elucidated [87]. SARS-CoV-2-specific antibodies have been demonstrated to wane within months [38,88] although a population-based study in Iceland showed SARS-CoV-2 antibodies did not decline within four months after infection [89]. However, the absence of specific antibodies in the serum does not mean an absence of immune memory, since cellular responses are of key importance in this context. Even if antibody titres decay over a few months or more to undetectable levels, the B cells that manufacture them may still be present, and there is strong evidence that T cells play a major role in combatting the virus [89]. Studies suggest that individuals with mild SARS-CoV-2 infection have a different pattern of T cell responses compared to those with more severe infection [90]. T cells active against SARS-CoV-1 have been detected 17 years post infection [91].

Reinfection has been hypothesized as occurring because of a short-lived high titre antibody response after the first infection but given the paucity of well-documented cases [92], little is understood about rates of reinfection at present, although more data are emerging both for vaccinated and unvaccinated individuals [77]. The pandemic is still in its early stages, so over the coming year, reinfection rates are likely to rise and will, therefore, yield more precise information on the duration of immunity both from infection and vaccination [93].

One important aspect in data collection associated with antibody response and its relationship to the probability of reinfection is the wide range of serological testing platforms used globally. It is, therefore, difficulty to compare results from one assay to another. For example, antibody reactivity to nucleocapsid protein indicates previous exposure to SARS-CoV-2, but not whether antibodies that can block infection (anti-spike protein antibodies) are present [94]. Also, antibody levels are highly dependent on the timing after exposure. A key goal for future studies is to ascertain the level and specificity of antibody to spike protein at the time of reinfection, to better understand immune correlates of protection.

The question of whether immunity generated by past infection (or indeed vaccination) prevents transmission from those who are reinfected is clearly important. The cycle time (*C_t_*) value of PCR diagnostic methods correlates with viral load, and low *C_t_* values (high viral load) might indicate infectiousness of the individual. Although *C_t_* values can vary substantially between various tests and laboratories, in one study, samples with *C_t_* values greater than 35 were only 8% positive for cultivable virus. A better proxy for infectiousness can be obtained through viral plaque assays that measure the amount of infectious virus. However, these assays require biosafety level 3 facilities and are labour intensive. As such the assays are not routinely performed in clinical laboratories.

### 4.8. Where do people acquire infection and who infects whom?

Epidemiologists employ three key methods in the study of transmission from person to person: household studies, contact tracing and whole-genome sequencing (WGS) of viral isolates (to ascertain who infects whom). A recent review of UK and other HIC data from all three approaches has been completed [95].

Where people acquire infection depends on many factors, including social distancing measures in place at any point in time in a given setting, prevailing compliance patterns to such measures which have varied over time in many countries as people's resolve wanes, variation between geographical locations, and confounding variables such as age, gender and cultural differences between social or ethnic groups within a defined population.

A visual representation of the public response to government directives specified under various tiers of constraints and social distancing rules (such as in the UK) in various locations, is provided by Google mobility data [96]. Google tracks mobile phone locations, thereby illustrating movement trends across different categories of places such as home, public transport, supermarkets, workplaces, retail locations and parks. An analysis of January to August 2021 data suggested that over a period covering full lockdown, through a phase of gradual relaxation of social distancing measures, to their complete removal in England on the 19 July, behavioural change was roughly linear, likely due to people anticipating future changes in social distancing regulations (electronic supplementary material, figure S4). Mobility data provide a very good indicator of how, by comparison with baseline (no social distancing regulations), average behaviour changes in defined populations. This, in turn, provides a crude guide to the extent to which *R*, which is greatly influenced by human mixing and movement patterns, may be impacted by social distancing measures (i.e. a 50% reduction from baseline for a given month could, optimistically, reflect a 50% reduction in the magnitude of *R*).

At the time of writing, mid-2021, contact tracing data in Europe and North America remain surprisingly limited. In part, this is a consequence of continued extensive spread of the virus. The test, trace and isolate (TTI) system as a tool to control spread, and as a source of epidemiological information, has limitations when viral spread is extensive, given the enormous workload involved in tracing contacts. It is best employed to control transmission during phases when rates of infection are low, as well illustrated in many East Asian countries such as Taiwan [23]. A good illustration of the challenges in contact tracing is illustrated by data from the first week of November 2020 in the UK. Of those estimated by the Office of National Statistics (ONS) to have been infected that week, only 30% were reached to ask for information on close contacts, and only 18% of the estimated close contacts of those newly infected that week were asked to self-isolate. Perhaps only 9% of these contacts did so fully, due to reported poor compliance in certain demographic and social groups. These numbers are clearly far too low to have a major impact on viral transmission or to provide a representative national picture of spread and control measure impact. Hopefully, contact tracing will play a significant role in identifying local outbreaks once mass vaccination creates sufficient herd immunity to greatly reduce transmission.

**Figure 6.**
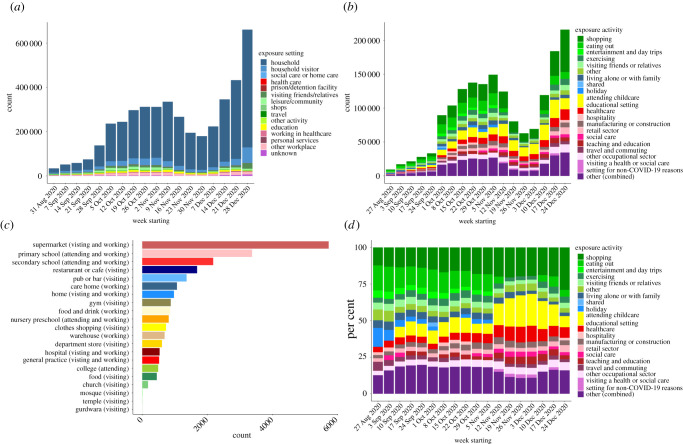
Exposure settings as documented in contact tracing data compiled by Public Health England, August–December 2020. All data show categories where exposure to infected contacts occurred in England by week and do not infer transmission. (*a*) Contacts by exposure setting. These are the settings where a person who tested positive for SARS-CoV-2 reported that they met with and potentially exposed their contacts (forward contact tracing). Work is ongoing to link contacts to future cases and to determine where transmission occurs. (*b*) Locations reported by people who tested positive for SARS-CoV-2 as possible exposure settings, defined as locations visited in the 3–7 days prior to symptom onset, or test date if asymptomatic (backward contact tracing). (*c*) Common locations reported by people who tested positive for SARS-CoV-2. Two or more individuals who tested positive reported the same location, defined by the same postcode, as a possible exposure setting in the 3–7 days prior to symptom onset, or test date if asymptomatic (backward contact tracing). Information on this type of event and the location are recorded but not information on contacts. (*d*) As (*c*) but with relative frequency of reported settings on the *y*-axis.

Data collected on a national scale by Public Health England (PHE) provide an interesting snapshot of the variables influencing infection acquisition in one time period as illustrated in figure 6.

The data reveal households are an especially important exposure setting. Of greater significance, however, is the question of where did the individual who seeded the household (the index case), acquire their infection? Figure 6*b* shows a wide variety of settings of importance in England, with supermarkets showing significant association. This ranking is likely to change both temporally and geographically, depending on seasonality and current social distancing restrictions in place.

Equally important to transmission is how different sectors or stratifications of populations or societies mix. This could be by age (the so-called WAIFW (who-acquires-infection-from-whom) matrices in infectious disease epidemiology [95,97–99]), or other factors. The intragenerational structure of households is also important. It can be strongly influenced by ethnicity and culture, and economic factors.

WGS has been used extensively to identify who infects whom for a variety of other viral infections, including HIV-1 [11]. Recently, it has been employed in elucidating the emergence of new SARS-CoV-2 variants by the UK COG sequencing consortium [100,101]. Much detail has emerged on the genotypic changes but much less is understood about the phenotypic impact of the recorded mutations. Much more attention must be directed toward these phenotypic changes

### 4.9. Mathematical models of virus transmission

Various types of models have been used to estimate the reproduction number *R_t_* of SARS-CoV-2 in the UK [11]. We focus here on models that have been used to inform government decision-making throughout the COVID-19 pandemic in the UK. These models have all been used in the same context and have repeatedly been compared against each other. A more in-depth review of SARS-CoV-2 transmission models used in the UK has been published previously [11]. An extended review of models used globally has been published by Xiang *et al.* [102]. Broadly they can be subdivided into statistical models that estimate *R_t_* by correlating the infection rate to selected covariates, such as the contact rate or individual mobility data, renewal equation models that estimate *R_t_* directly from an incidence time series, and mechanistic models that fit a transmission dynamics model to incidence data and determine *R_t_* from the estimated parameter values. A hybrid approach where changes in mechanistic model parameters over time are estimated from relevant covariate data using regression techniques has also been employed.

The various models used to predict how *R_t_* changes dependent on certain variables such as the environment, interventions and changes in human behaviour, have advantages and disadvantages and are, therefore, complementary. Predictions may be inaccurate due to unexpected changes in individual variables, or in the relationship between these variables and *R_t_*. Renewal equation models are good at detecting instantaneous changes in *R_t_* from incidence data but they do not consider the underlying reasons for changes in *R_t_*. They, therefore, cannot be used to distinguish or predict how changes in epidemiological variables affect the value of the composite parameter. Depending on their level of detail, mechanistic models can be used to evaluate how changes in interventions and epidemiological variables affect *R_t_* and to synthesize different data sources. However, because they can incorporate more details than other model types, they require a greater variety of high-quality data sources to inform model assumptions and estimate many parameters concomitant with increased complexity. New areas of SARS-CoV-2 research that have benefited from mechanistic models are the design of vaccination strategies [103,104] and the investigation of the properties of new SARS-CoV-2 variants [105–107].

Problems for decision-makers can arise when different models produce different estimates. Some differences are easy to explain, for example, different input parameter values. However, differences due to model structure are more difficult to understand and require a systematic comparison. Even models of the same type, for example, stochastic individual-based transmission dynamic models, vary in the number and type of compartments they contain. It is not always clear how these differences will influence parameter estimates derived by Bayesian fitting procedures.

All models used in the early phase of COVID-19 in the UK and reviewed in [11] use probabilistic methods and sensitivity analyses to construct uncertainty bounds around *R_t_* estimates. More complex models with a greater number of parameters are more prone to overfitting and hence underestimating the uncertainty in their estimates. Another factor that influences the uncertainty around *R_t_* is the timeframe of estimation. Longer time frames result in narrower uncertainty bounds. The most important factor influencing the accuracy of *R_t_* estimates is the timeliness and quality of input data streams.

Much recent work on models has focused on the impact of vaccination, and the associated creation of herd immunity, given the licensing of many vaccines and their subsequent roll out in different countries in late 2020 [104,108,109]. The transmission environment is highly heterogeneous and influenced by many time-sensitive factors, such as evolving herd immunity created both by vaccination and past infection, to continuous and sometimes seasonally related mobility and mixing patterns, and the changing viral variant composite within a country. These sources of uncertainty will need addressing in the future. As most epidemic models rely on average contact patterns between groups and average parameter values for key epidemiological processes such as incubation and infectious periods and generation times, they cannot easily account for these many sources of heterogeneity. As such, the derived predicted patterns of infection have considerable uncertainty bounds.

### 4.10. Creation of herd immunity by mass vaccination

**Figure 7.**
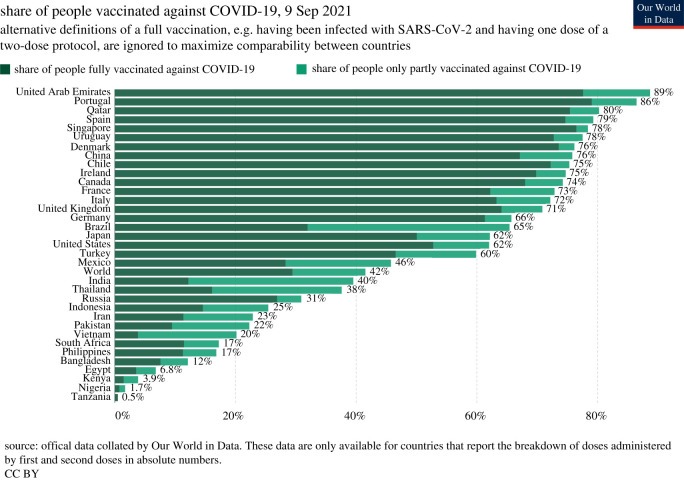
Percentages of populations vaccinated against SARS-CoV-2 infection in a selection of countries. The data are only available for countries that report the breakdown of doses administered by first and second doses in absolute numbers (source: Our World in Data. Data as of 19 August 2021 [110]).

Since the licensing and large-scale manufacture of a number of vaccines protecting against SARS-CoV-2 infection, many HIC have seen significant changes in both morbidity and mortality. The most vulnerable groups were immunized first along with front-line healthcare staff, those in care facilities, and those with other risk factors. Coverage with two doses of the most widely used vaccines (Astra Zeneca, Moderna, Pfizer/BioNTech, Johnson & Johnson, Sinopharm, Sinovac, Sputnik V) in the world reached high levels by 16 August 2021 (figure 7). As of 20 August 2021, estimates suggest that 32.2% of the world's population have received at least one dose, and 24.2% are fully vaccinated [110]. Roughly 4.9 billion doses have been administered globally and 36 million are currently administered each day. However, only 1.3% of people in low-income countries have received at least one dose [110]. There is a close positive correlation between percentage vaccine coverage and GDP per capita of a country. Given the movement patterns of people prior to this pandemic, SARS-CoV-2 will continue to circulate. Even countries closing their borders could not completely avoid repeated reimportation. It is important to recognize that the net rate of viral evolution is proportional to the number of new infections globally per unit of time, so all countries have a vested interest in ensuring the whole world receives adequate vaccine supplies.

The build-up of herd immunity by mass vaccination depends on five factors. These are the magnitude of the basic reproductive number (*R*_0_), vaccine efficacy (both in terms of preventing infection and in terms of reducing transmission following vaccine break-through infections), public acceptance of the vaccine (high coverage is required to take *R*_0_ < 1), the supply chain of vaccines and the logistics of delivery (cold chains) and the rate at which populations can be vaccinated. With the possible exception of city states, even in most countries with good healthcare infrastructures, it will take an estimated 12 months to cover the entire adult population and teenagers. Of concern is the viral evolution of variants against which current vaccines have lower efficacy. Current manufacturing platforms developed for SARS-CoV-2 vaccines should permit rapid adjustments to be made in response to evolutionary events, as is the case for influenza A vaccines. This will require continual worldwide surveillance of viral evolution. Longevity of vaccine efficacy has yet to be evaluated, as those currently available have not been in use for long enough to determine if protection against either infection of serious disease lasts beyond 6–12 months. Most manufacturing companies plan to produce booster doses for deployment in selected countries in autumn 2021 [27]. This is controversial, given that many LMIC have yet to receive adequate supplies to first vaccinate even their high-risk populations. Israel is already offering booster shots, given concerns about vaccine immunity duration [111].

**Figure 8.**
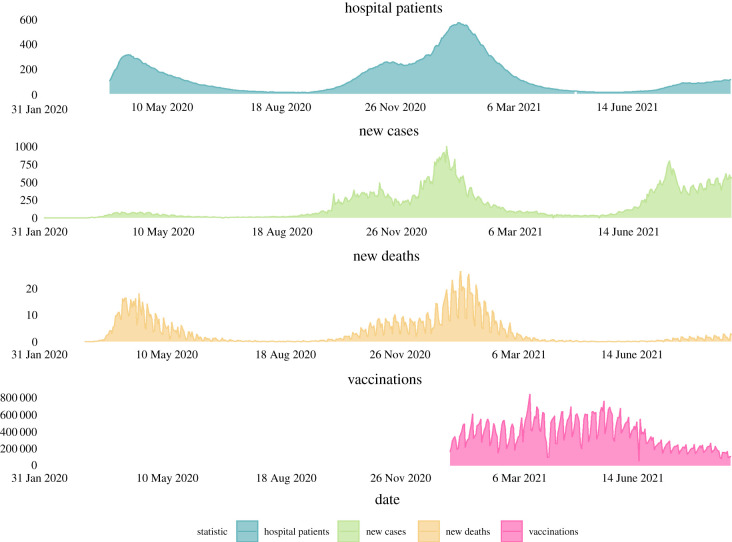
Case reports of SARS-CoV-2 infection, hospitalizations resulting from infection, deaths reported due to COVID-19 and vaccinations per day in the United Kingdom from the start of the epidemic to 8 September 2021 (excepting hospitalizations where data only began being reported in late March 2020; there was only targeted testing for COVID-19 cases up to late May 2020 in the UK, before wider testing gradually became available) (source: Our World Our Data [112]). The data illustrate how the relationship between the three indicators of impact (cases, hospitalizations and deaths) becomes uncoupled following mass vaccination (0.6% of UK population fully COVID-19 vaccinated 10 January 2021; 64% fully vaccinated 5 September 2021 [112]). Note: *y*-axis for hospital patients, new cases and new deaths are per million. Vaccination numbers are actual number received.

The early impact of mass vaccination, when high coverage levels are achieved (greater than 90%) in the most vulnerable age groups, is demonstrated by comparing daily reported cases, hospitalizations and deaths due to COVID-19 in the UK prior and post mass vaccination (figure 8). Cases remained high in mid-2021 in the UK, especially in younger, non-immunized age groups. Hospitalizations were lower than during previous phases of high infection levels, but not markedly so. COVID-19-related deaths concurrently fell, but not as yet to very low levels (running at about 100 deaths per day mid-August 2021).

The history of mass vaccination demonstrates what an important contributor it has been to the health and welfare of the world's population since its introduction in the 1960s. Medical historians advocate it to be the most important intervention in globally reducing years of life lost, alongside antibiotics. It is important to note that only the smallpox virus [113] has been eradicated by mass vaccination to date, despite collaborative international efforts targeted at childhood viral infections such as polio, measles, mumps and rubella [114]. It seems likely that SARS-CoV-2 will remain endemic within the world with seasonal boom and bust patterns of incidence, like other common respiratory viral infections such as influenza A and B, rhinoviruses, RSV and other coronaviruses.

The requirements for creating sufficient herd immunity to stop transmission is well understood from the theory surrounding mass vaccination and its impact on disease epidemiology [115]. For a perfect vaccine with 100% effectiveness (in terms of preventing infection as well as transmission) and lifelong duration of protection against disease, and infection that generates infectiousness, the fraction in a population who must be immunized to totally block transmission, *p*, is: *p* > [1 − 1/*R*_0_]. If the vaccine has an effectiveness *ε* (defined as proportion protected against disease and infection that results in onward transmission), then *p* > [1 − 1/*R*_0_]/*ε* is required to block all transmission. As a specific example, for an *R*_0_ value of 2.5 and efficacy of 70% (*ε* = 0.7), implies that coverage *p* must be greater than 86%. If effectiveness is 95%, coverage required reduces to just over 65%. For the now widespread Delta variant, with *R*_0_ = 5–6 and vaccine effectiveness, *ε* of 80%, then the required coverage (using *R*_0_ = 5.5) becomes greater than 100%. Vaccination alone will, therefore, not eradicate the virus from the world.

The current portfolio of vaccines in mass use is summarized in table 3. Reported efficacy values range from 51% to 95%. The definition of efficacy is important and in the phase III trials for COVID-19 vaccines has been based on preventing symptomatic infection of any severity [143]. Real-world experience of vaccines over the first half of 2021 suggests that defining efficacy in different ways will become more important for those fully vaccinated who acquire infection, such as the prevention of serious disease, onward transmission, hospitalization and death. No protection duration data are available currently, since detailed phase IV studies are required of a large sample of vaccinated patients, with follow-up over many years. Reinfection rates in countries with high coverage such as Israel, suggest full protection wanes within 12 months for some. However, it is still unclear if all vaccines that are currently widely used protect against severe disease or reduce transmission of infection [144]. As noted earlier a recent survey suggests that those who acquire infection even if fully vaccinated may have similar viral loads of the Delta variant than those infected but not vaccinated [35]. A small study involving only a few patients found a similar pattern with those vaccinated but infected with the Alpha variant [145]. Better understanding these issues, variant by variant, is urgent. It will help countries decide if repeated vaccination is required year by year with modified booster shots designed to protect against a range of viral variants. Most vaccines in use seem to offer good protection against serious disease arising from infection.

**Table 3.** Vaccines available mid-2021 for country-wide mass vaccination programmes (reference number in brackets) with reported efficacies from clinical trials and from real-world studies in vaccinated populations.

vaccine	type	publications in peer-reviewed literature	in use	interval between two doses	cost	temperature for storage	efficacy against infection from phase 3 trials		efficacy against infection from real-world data		efficacy against severe disease/hospitalization
							after 1st dose	after 2nd dose	after 1st dose	after 2nd dose	
Oxford-Astra Zeneca	viral vector	yes [116]	yes	Up to 12 weeks	US $4	2–8°C	76% [117]	82.4% [117]	60–70% [118]	unknown	94% [119]
Moderna	RNA	yes [120]	yes	28 days	US $33	−20°C (but at 2–8°C for 30 days) [121]	80.2% [122]	94.1% [120]	80% [123]	90% [123]	96% among adults aged ≥65 years [124]
Pfizer-BioNTech	RNA	yes [125]	yes	21 days	US $20	−70°C (but at 2-8°C for 30 days) [126]	52% [125]	95% [125]	85% [116]	93.7% [127]–95.3% [116]	97.2% [116]
Sinopharm	inactivated	yes [128]	yes	21 days	unknown	2–8°C	unknown	78.1% [129]	unknown	90% [130]	79% [131]
Sinovac	inactivated	yes [132]	yes	14 days	US $29.75	2–8°C	unknown	50.65% [133]	unknown	unknown	85%–100% [134,135]
Sputnik V (Gamaleya)	viral vector	none identified	yes	1 dose suggested	US $10	2–8°C	73.1% [136]	91.6% [136]	unknown	97.6% [137]	unknown
Novavax	nanoparticle	yes	no	21 days	US $16	2–8°C	83.4% [138]	89.7% [138]–96.4% [139]	—	—	100% [138]
Johnson & Johnson	viral vector	yes	yes	1 dose suggested	US $10	9–25°C	66.1% [140]	—	76.7% [141]	—	100% [140]
CanSino	viral vector	yes	yes	1 dose suggested	unknown	2–8°C	65.7% [142]	—	unknown	unknown	91% [142]

If the immune response arising from vaccination behaves similarly to natural infection, it is likely that protection may be limited to about a year. As such, repeated vaccination will be required, as is the case for influenza A. Whether a short duration of immunity following natural coronavirus infection is due to continued viral evolution such that ‘escape’ mutant variants are selected for over time, or it lies in the ability of the virus to modulate or evade the effectiveness of the immunity generated by natural infection, is unclear for SAR-CoV-2 at present.

Much focus, recently, has been placed on how well the different vaccines protect against a range of viral variants. A good example is provided by a recent study of the Moderna vaccine. Mutations in the SARS-CoV-2 genome are thought to diminish vaccine-induced protective immune responses as antibody titres wane over time. The recent assessment of SARS-CoV-2 variants B.1.1.7 (Alpha), B.1.351 (Beta), P.1 (Gamma), B.1.429 (Epsilon), B.1.526 (Iota) and B.1.617.2 (Delta) on binding, neutralizing, and ACE-2-competing antibodies elicited by the vaccine mRNA-1273 over seven months yields some encouraging results [146]. Cross-reactive neutralizing responses were rare after a single dose. At the peak of responses to the second vaccine dose, all individuals had responses to all variants. Binding and functional antibodies against variants persisted in most subjects for six months after the primary series of the mRNA-1273 vaccination. Across all the assays reported, B.1.351 (the Beta variant) had the lowest antibody recognition. This publication is one of a wide variety of ongoing studies employing a wide range of the current vaccines to assess the need for booster vaccinations as captured in table 4, where it is clear that much remains to be done in this area.

**Table 4.** Published information on the impact of licensed vaccines against SARS-CoV-2 infection and associated disease in protecting against infection and disease from different viral variants.

vaccine	efficacy against transmission from phase 3 trials		effective or not against infection of different variants							
	after 1st dose	after 2nd dose	variants of concern (VOC)				variants of interest (VOI)			
			B.1.1.7 (Alpha)	B.1.351 (Beta)	P.1 (Gamma)	B.1.617 (Delta)	B.1.525 (Eta)	B.1.526 (Iota)	B.1.617 (Kappa)	C.37 (Lambda)
Oxford-Astra Zeneca	67% [117]	50% [117]	yes (70.4% [147]–74.5% [127])	no (10.4%) [148]	unknown	yes (67%) [127]	unknown		yes	unknown
Moderna	≥61% (estimate) [149]	unknown	unknown	remains to be determined [150]	unknown	yes [121]	unknown		yes [121]	unknown
Pfizer-BioNTech	unknown		yes (89.5%) [151,152]	yes (75%) [152,153]	unknown	yes (64% [154]–88% [127])	unknown	yes [153]	unknown	
Sinopharm	unknown		yes [155]	yes [156]	unknown					
Sinovac	unknown		yes [157]	yes [157]	no [100]	unknown				
Sputnik V (Gamaleya)	unknown		unknown			yes (90%) [158]	unknown			
Novaxvax	unknown		yes (86.3%) [138]	yes (49.4–60%) [139,159]	unknown					
Johnson & Johnson	74% [140]	—	unknown			yes [160]	unknown			
CanSino	unknown		unknown							

A number of studies have examined how duration of protection impacts the level of herd immunity required to eliminate viral transmission, and what subsequent key policy objectives of mass vaccination should be [104]. These studies were initiated before the Delta variant emergence, and their relevance is, therefore, less clear given the high *R*_0_ of this variant (*R*_0_ = 5–6, requiring 100% vaccination coverage to block transmission, as explained earlier). Given so-called vaccine hesitancy in many western societies, achieving this goal is currently unlikely [161–163]. Achieving high coverage will require good government messaging to the public and may also require mandating vaccination for many activities such as workplaces, schools and hospitals or indeed any crowded space. Already activities such as air travel or entry to a country require evidence of vaccination in the form of vaccine passports [164]. Discussions of these sorts of actions are likely to become more intense as mass vaccination progresses and the incidence of infection declines but is never zero [104,161].

Given that transmission elimination of COVID-19 is virtually impossible, except in very small countries where stochastic effects may cause extinction, arguably the primary objective of mass vaccination should be preventing hospitalization and death. This leads to the policy question of how does this impact who to prioritize for vaccination? Two policy options require consideration: namely, reducing net mortality due to infection as quickly as possible (within the first year of mass vaccination), or minimizing years of life lost due to infection in the longer term. A more complex second objective could be years of healthy life lost, given the growing evidence that in some people the viral infection induces long-term health impact (known as long COVID [165–167]). Calculating years of life lost requires data on demography (numbers in different age classes) and epidemiology (age-dependent CFR or IFR) as depicted in Supplementary Material, Figure S3. Recent analysis of this issue based on HIC demography reveals that the two options end up coming to the same solution; namely, start in the oldest age groups (80+ years of age) and move to progressively younger groups as vaccine supply builds, to both minimize mortality most efficiently and minimize total years of life lost [108]. This is because the CFR rises so steeply with age for SARS-CoV-2 infection (Supplementary Material, Figure S3), thus although forming a small proportion of the total population, the elderly contribute most to net overall mortality [108].

For other countries with a more J-shaped curve of numbers per age class, such as some LMIC, the calculations remain to be done, as there is an absence of good age-structured CFR estimates and poor reporting of the cause of death of the elderly.

## 5. Conclusion

A large volume of research over the past 18 months has provided a much-improved understanding of the key processes that determine the transmission dynamics of SARS-CoV-2. Despite this rapid output of scientific research, important gaps remain in our understanding of the epidemiology of this virus and the pathogenesis it induces. The key challenges for the coming year are to fill these gaps as rapidly as possible, using all the modern and traditional methods of infectious disease epidemiology.

Epidemiological gaps include the following. There is a need to expand the use of viral WGS to understand more about which variants are in circulation, who infects whom, the precise chains of transmission and the seeding of household infection [168]. This will remain important, as vaccine coverage over the coming 12 months rises (provided supply and delivery meet policymakers' expectations), and the chains of transmission in those still susceptible to infection need to be extinguished.

Understanding how important asymptomatic infections are to sustaining transmission must be improved. What factors predispose to asymptomatic infection besides age? What are the incubation and infectious periods of asymptomatic people? Are these different from symptomatic individuals and, if so, by what degree? What predisposes to ‘long COVID' disease and does the virus persist in the body for long periods post recovery from acute symptoms of infection? At present long-term viral persistence seems unlikely, but much more attention needs to be paid to what factors are associated with persistent symptoms over many months. All of these major epidemiological variables may vary between viral variants.

Contact tracing via schemes such as the TTI programme in the UK, need to embrace modern data capture technologies, taking due note of individual data protection regulations. However, where mobile phones can be used to track infected people and their contacts, more effective systems for tracing and contact isolation can result [169]. As we move into a period of mass vaccination, the issue of what is the typical duration of immunity to reinfection, in both those recovered from natural infection, and those vaccinated, is of foremost importance. Across the licensed vaccines, and against the different variants in circulation, does the efficacy and duration of protection vary? Linked questions include are those vaccinated but who acquire infection infectious to others, and how long do markers of protection last (both antibodies and T cells specific to viral antigens)? What are the best correlates of protection against infection is always an important question, but a difficult one to answer.

The greatest area of uncertainty concerns to what extent the observed, continuous genotypic changes in the viral genome translate into phenotype changes. Our understanding is growing but still limited. More effort needs to be applied to linking some clearly defined phenotypic and epidemiological variables to the genome data in the sequencing databases. This is not easy, since longitudinal follow-up is required of the patients from which viral samples are collected. This area is of high importance, given the strong selective pressure that will be applied on the virus by mass vaccination.

A global effort in phase IV studies of individuals who have been vaccinated must be conducted for each of the vaccines in widespread use. It seems probable at present that immunity duration post infection may be a year or so given studies of other coronaviruses. Some vaccines may do better, but it seems likely that vaccination will have to be repeated for any given individual, yearly or hopefully less frequently. Viral evolution globally will play a significant role in this aspect of mass vaccination.

Although great strides have been made in data capture of case reports, hospitalizations and deaths associated with infection, there is still much room for improvement in many countries where healthcare infrastructure and resources are limited. Vaccination rollout will stretch these resources further and hence much more support is needed in LMIC from HIC over the coming year to ensure the creation of herd immunity by mass vaccination is a global target, not just one for the industrialized nations. The unit for assessing success in the creation of herd immunity by vaccination is the world, not any individual country.

## Data accessibility

All data used in this manuscript are secondary and can be located through the references provided.
